# A Phthalocyanine Optical Probe Responding to Cationic Surfactants Containing Long Carbon Chains with High Selectivity in Total Water Phase and Its Applications

**DOI:** 10.3390/molecules30214184

**Published:** 2025-10-26

**Authors:** Yan Zhang, Tao Zhou, Yabin Deng, Xiao Zheng, Jiaqi Guo, Ping Huang, Donghui Li

**Affiliations:** Cancer Research Center, School of Medicine, Xiamen University, Xiamen 361102, China; zhangyan51168@163.com (Y.Z.); zeltar@163.com (T.Z.); dyb@xmu.edu.cn (Y.D.); gjqdyx@xmu.edu.cn (J.G.)

**Keywords:** phthalocyanine, cationic surfactant, optical probe, association

## Abstract

The analysis of cationic surfactants with high selectivity is a source of great research interest. In this study, the absorption spectra of tetra-sulphonated metal phthalocyanine (coordinated by iron, zinc, cobalt, and nickel) in the presence of cationic surfactants in complete aqueous solutions were investigated. Interestingly, the absorption spectra of tetra-sulphonated nickel phthalocyanine (NiS_4_Pc) exhibits a remarkable response to the cationic surfactants compared with other water-soluble metal phthalocyanines. Further investigation has yielded important findings that cationic surfactants with carbon chains containing twelve or more carbons cause distinct spectral responses, and the response behaviors are highly similar, showing a typical structure–activity relationship. Studies on the mechanism of response indicate that the spectral behavior could be attributed to the dramatic binding effects of structure-matched cationic surfactants on the self-association equilibrium of nickel phthalocyanine. Based on the above findings, we applied NiS_4_Pc as a directly responsive optical probe for the quantitative analysis of long carbon chain cationic surfactants. Due to the high degree of similarity in optical responding, this method can be used to determine the single cationic surfactant and the total cationic surfactants. It is worth mentioning that NiS_4_Pc is a water-soluble optical probe that can be used in a complete aqueous phase. Therefore, this method is not only selective but also easy and fast to operate, without the need for organic solvents. Under optimized conditions, the average calibration curve equation of the method is y = 1.66 − 0.0173 x, r = 0.9987, with a limit of detection of 3 × 10^−6^ mol L^−1^. This method has been applied to the determination of real samples, for which we obtained satisfactory results. We not only describe the establishment of a new method for the direct quantitative analysis of cationic surfactants but also propose a new strategy for obtaining phthalocyanine-based optical probes in this study, which explored the novel application of phthalocyanine compounds in analytical sciences.

## 1. Introduction

Cationic surfactants have a bactericidal function because they can adsorb on the surface of negatively charged bacteria, increasing the permeability of the bacterial cell wall, thereby changing the components in the bacteria [1]. Cationic surfactants have also been used as metal corrosion inhibitors [2], supercritical carbon dioxide additives [3], skin care additives [4], biocides [5], water-treatment agents and algaecides [6], as well as others. They are widely used in medicine and daily chemical industries [7]. However, the use of cationic surfactants can also introduce pollutants into the environment [8]. Therefore, quantitative analysis of cationic surfactants is important work.

Common analytical methods for cationic surfactants include two-phase titration [9], HPLC [10], spectrophotometry [11], fluorimetry [12], polarography [13], and thin-layer chromatography [14]. Some of these methods are complicated to operate, and some require the use of large amounts of organic solvents or large-scale instruments. In addition, they can usually only be used for the determination of a single cationic surfactant. As a result, it is of great practical significance to establish an easily operated quantitative analysis method for cationic surfactants for their direct measurement in the total aqueous phase.

Phthalocyanines are macrocyclic structures formed by four iso-indole units connected by four nitrogen atoms; the center can be combined with most metal elements, forming metal phthalocyanines [15]. Due to their excellent optical performance, metal phthalocyanines have a wide range of applications in many high-tech fields [16,17,18]. Because of their unique absorption characteristics in the long-wavelength region, metal phthalocyanines exhibit great potential for application in analytical sciences [19,20,21].

Metal phthalocyanines are prone to association in solution, which can lead to the formation of dimers. The optical properties of dimers are significantly different from monomers, which can have an important impact on the application of phthalocyanine compounds [22,23,24,25,26,27,28]. This is the reason why the study of phthalocyanine compound association has long been of concern. Research has shown that tetra-sulfonated metal phthalocyanine compounds mainly exist in monomeric form in organic media and have a strong tendency for self-association in aqueous media. The formation of association complexes leads to significant changes in molecular spectral properties such as light absorption spectra, fluorescence quantum yield, fluorescence lifetime, and the shift in fluorescence emission [29,30]. Research has shown that the presence of surfactants has a significant influence on the dimerization of sulfonated metal phthalocyanines [31]. For example, the aggregation behavior of tetra-sulfonated zinc phthalocyanines was obviously influenced when cationic surfactants were present in aqueous solution [32]. We also found a similar phenomenon on tetra-sulfonated nickel phthalocyanine in this study.

In this study, we found that the self-association of tetra-sulphonated metal phthalocyanines occurred in aqueous solution, and their association equilibrium was influenced in the presence of cationic surfactants, especially in the case of tetra-sulfonated nickel phthalocyanine. Its change in the absorption spectra was significantly correlated with the length of carbon chains in cationic surfactants, showing a typical structure–activity relationship. Based on these two important findings, we have established a new method for the direct analysis of cationic surfactants tailing a long carbon chain using NiS_4_Pc as a highly selective optical probe. The reaction mechanism was investigated and discussed. The present method is not only selective and easy to operate but also environmentally friendly because it can be directly performed in a completely aqueous phase without any use of organic solvents.

There are few reports on the direct determination of surfactants in a completely aqueous phase employing phthalocyanines or their analogs, porphyrins. Cobalt phthalocyanine was used as a novel molecular-recognition reagent for the determination of anionic surfactants [33]. Two porphyrin compounds were used for the analysis of cationic [34] and non-ionic surfactants [35]. To our knowledge, the use of tetra-sulfonated metal phthalocyanines as optical probes for the direct determination of cationic surfactants containing long carbon chain has not been reported.

## 2. Results and Discussion

### 2.1. Molecular Structure and Spectral Characteristics of Tetra-Sulphonated Metal Phthalocyanines

**Figure 1:**
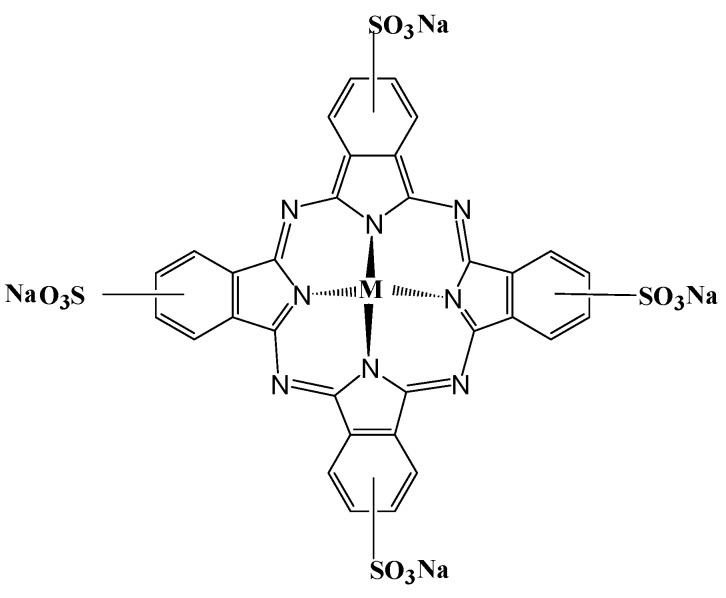
Molecular structure of tetra-sulphonated metal phthalocyanines (MS_4_Pc, M = Fe, Co, Ni, and Zn).

**Figure 2:**
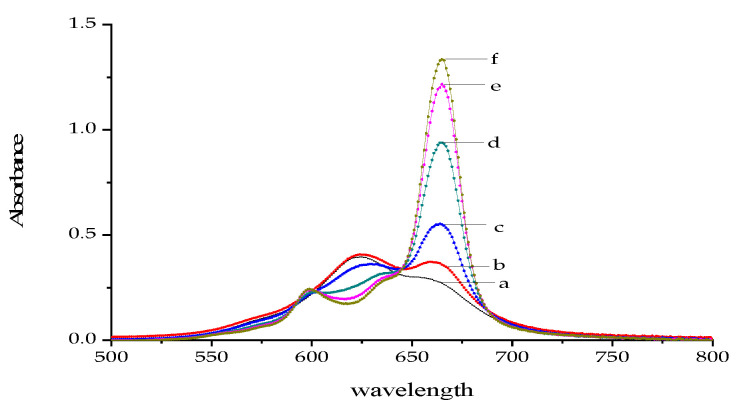
Absorption spectra of NiS_4_Pc in ethanol–water phase. The concentrations of ethanol (*v*/*v*, a–f) were 0%, 10%, 20%, 30%, 40%, 50%, and 60%, respectively. [NiS_4_Pc] = 20.0 μM.

The parent structure of metal phthalocyanines, which is also called azaporphyrin, has a similar structure to that of porphyrin. For the tetra-sulfo-substituted metal phthalocyanines, there are four strongly polar, negatively charged sulfonic acid groups on each peripheral benzene ring (Figure 1). This gives it excellent water solubility and advantages for use in the aqueous phase. Self-association occurs easily for phthalocyanine compounds, and this type of interaction is well-suited for investigation using molecular spectroscopy [36]. This phenomenon was also confirmed in NiS_4_Pc by the following study, in which, as different concentrations of ethanol were added to an aqueous solution of NiS_4_Pc, absorption of the dimer was found to be gradually decreased, and the absorption of the monomer was gradually enhanced (Figure 2) due to dissociation by organic solvent, which indicates that NiS_4_Pc has a strong self-association tendency in the aqueous solution.

**Figure 3:**
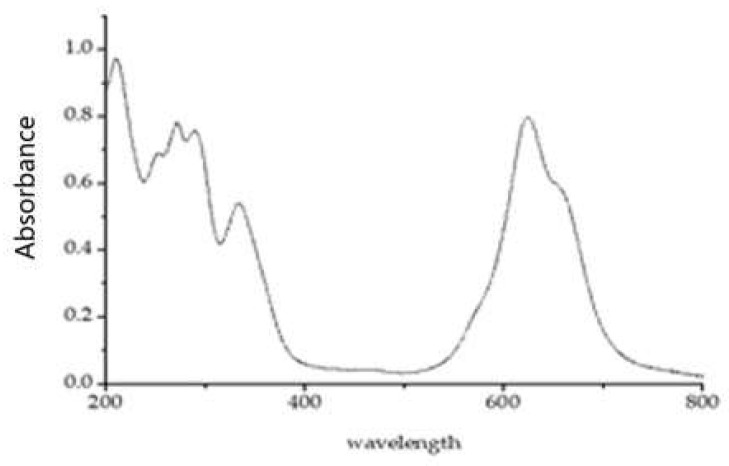
Absorption spectrum of NiS_4_Pc (aqueous solution), [NiS_4_Pc] = 20.0 μM.

In most cases, the absorption spectrum of a phthalocyanine compound can be divided into two regions (Figure 3): a short-wavelength region (called the B-band) and a long-wavelength region (called the Q band). Because the Q band is sensitive to the association of metal phthalocyanines, we focused our discussion of the spectral behavior on this region.

### 2.2. Discussion on Reaction Mechanism

**Figure 4:**
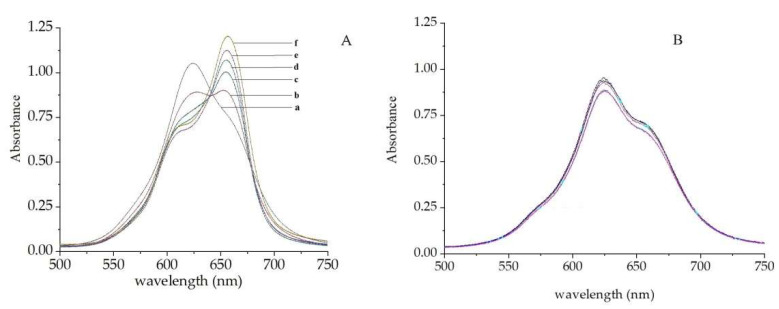
Absorption spectra of NiS_4_Pc in the presence of different concentrations of CPC (**A**) and BTBA (benzyltributylammonium chloride) (**B**). The concentrations of CPC (a–f) and BTBA are 0, 10.0, 20.0, 30.0, 40.0, and 50.0 μM, respectively. [NiS_4_Pc] = 20.0 μM.

As described above, phthalocyanine compounds tend to be self-associated in an aqueous solution, in which monomers and dimers of metal phthalocyanines (such as NiS_4_Pc) co-exist. We observed that in the presence of cationic surfactants with a long carbon chain containing twelve or more carbon atoms, the absorption peak of the monomer species of NiS_4_Pc increased significantly, while the absorption peak of the dimer decreased significantly (Figure 4A). This spectral-changing behavior is similar to what is seen in the ethanol–water system. The absorption spectra of NiS_4_Pc showed an isobestic point when different concentrations of CPC (cetylpyridinium chloride) were present, indicating that there were different species (monomer and dimer) of NiS_4_Pc in the aqueous solution, and the association equilibrium between the monomers and dimers shifted in the presence of CPC.

The parent structure of NiS_4_Pc has a hydrophobic, large π-conjugated system and a planar structure with four negatively charged sulfonic acid groups on the peripheral benzene rings. Such a structure allows NiS_4_Pc to electrostatically combine with positively charged cationic surfactants. In addition, the hydrophobic plane of phthalocyanine can interact with the hydrophobic carbon chains of surfactants by a hydrophobic interaction, forming a NiS_4_Pc–cationic surfactant associate. As a result of this interaction, the self-association equilibrium of NiS_4_Pc shifts, and the dimer dissociates to the monomer. However, further research revealed that not all cationic surfactants could cause such spectral changes (Figure 4B, and more information can be obtained in the Supplementary Material); only cationic surfactants with an alkyl carbon chain containing twelve or more carbons could bring about such an effect (Figure 4A). We believe that a cationic surfactant capable of shifting the self-association equilibrium of NiS_4_Pc should have a structure matching with that of NiS_4_Pc. The hydrophobic domain (carbon chain) of the cationic surfactant must be large enough to form a strong association with NiS_4_Pc, whereby the self-association equilibrium of NiS_4_Pc is weakened. This is manifested by an increase in the absorption peak of the monomer and a decrease in the absorption peak of the dimer.

A recently published paper about the influence of cationic surfactants with long carbon chains on the aggregation behavior of a water-soluble phthalocyanine, tetra-sulfonated zinc phthalocyanine (ZnS_4_Pc), by a combined experimental and computational study, reveals the very strong interaction between cationic surfactants and ZnS_4_Pc [32]. ZnS_4_Pc and NiS_4_Pc have the same parent structure, and both of their central coordinating atoms are divalent metal ions; therefore, there is reason to believe that the interactions between the two and long-chain cationic surfactants are very similar. That is to say, long-chain cationic surfactants also have strong interactions with NiS_4_Pc, leading to a shift in the association equilibrium of NiS_4_Pc in water. This latest report provides strong support for our discussion of the reaction mechanism.

The above discussion pointed out that NiS_4_Pc can selectively respond to cationic surfactants with matching structures.

### 2.3. Optimization of the Experimental Conditions

#### 2.3.1. Selection of Metal Phthalocyanine Compounds

**Figure 5:**
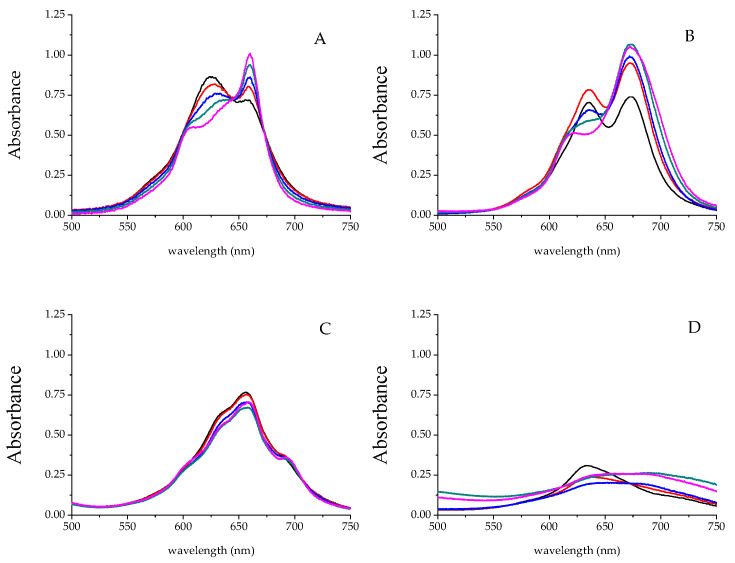
Absorption spectra of different water-soluble metal phthalocyanine compounds in the presence of increasing concentrations of CPC. The tetra-sulfonated metal phthalocyanine compounds (**A**–**D**) were NiS_4_Pc, ZnS_4_Pc, CoS_4_Pc, and FeS_4_Pc, respectively. The concentrations of CPC were 10.0, 20.0, 30.0, 40.0, and 50 μM, respectively. [NiS_4_Pc] = 20.0 μM.

The reactions of four kinds of tetra-sulfonated metal phthalocyanines (NiS_4_Pc, FeS_4_Pc, CoS_4_Pc, and ZnS_4_Pc), all water-soluble, with different concentrations of CPC were investigated (Figure 5). The results showed that there was no reliable regularity in the response of ZnS_4_Pc to CPC (Figure 5B), while CoS_4_Pc (Figure 5C) and FeS_4_Pc (Figure 5D) had less of a response to CPC. Interestingly, it was found that NiS_4_Pc could respond significantly to CPC and showed good regularity (Figure 5A). Therefore, NiS_4_Pc was selected as the optical probe for cationic surfactants in the following study.

#### 2.3.2. Effect of pH

The effect of reaction acidity on the linearity of the calibration curve for the determination of a cationic surfactant (CPC) was investigated, using a broad phosphate buffer system with pH 1.0–11.0 as the reaction medium. The results showed that the linear range and regression coefficients of the calibration curves were best in the medium with pH 2.0 (Table 1).

#### 2.3.3. Selection of Buffer Systems

The effects of three pH 1.0 buffers (hydrochloric acid, hydrochloric acid–potassium chloride, and phosphate buffer) on the calibration curves were investigated. The results showed that the composition of the buffers had little effect on the linearity and range of the calibration curves. A phosphate buffer with a pH of 1.0 was finally selected as the reaction medium.

#### 2.3.4. Effect of Ion Strength

The effects of ion strength on the calibration curves were investigated. It was found that NiS_4_Pc responds to cationic surfactant with good linearity at a lower ion strength. As the ion strength increases, the linear response behavior deteriorates, and there is no linear response in a high-ion-strength medium. Detailed information can be found in the Supplementary Material (Figure S1).

#### 2.3.5. Selection of Wavelength Pair for Measurements

It was found that the linear parameters of a calibration curve varied with the choice of wavelength pairs for measurements. Thus, calibration curves were constructed and compared by determining the absorbance ratios, using different wavelength pairs to obtain the best one. The following wavelength pairs were chosen and compared: 624 nm to 658 nm, 624 nm to 659 nm, 624 nm to 660 nm, 625 nm to 658 nm, 625 nm to 659 nm, 625 nm to 660 nm, 626 nm to 658 nm, 626 nm to 659 nm, and 626 nm to 660 nm. The best linear parameters were achieved by employing the pair of 625 nm to 659 nm, which was selected for the quantitative analysis in this study.

#### 2.3.6. Effects of Reaction Time and Temperature

**Figure 6:**
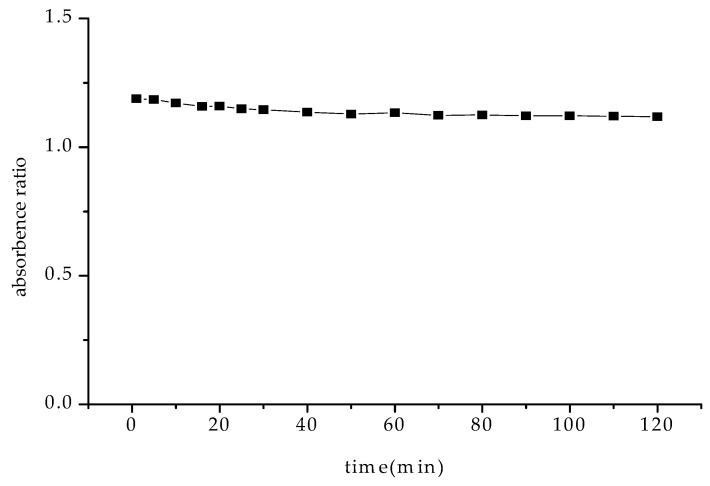
Effect of reaction time on the absorbance ratio. [NiS_4_Pc] = 20.0 μM.

**Figure 7:**
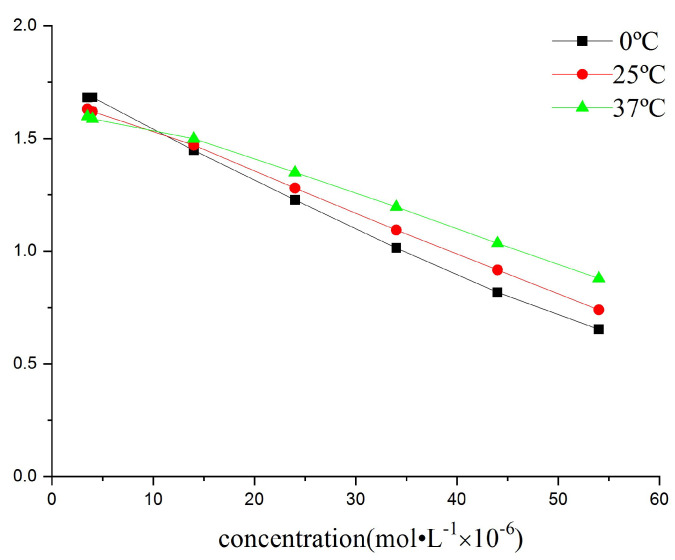
Effect of temperature on the CPC calibration curves. [NiS_4_Pc] = 20.0 μM.

The effects of reaction time and temperature on the absorbance ratios were investigated. Our investigations showed that the absorbance ratio did not change much with time (Figure 6). A time of 5 min was then set to perform a reaction. Upon studying the linearity at 0 °C, 25 °C and 37 °C (Figure 7), we found that it was better at 0 °C and 25 °C than at 37 °C, implying that for easy operation, measurements may be performed at normal atmospheric temperature.

#### 2.3.7. Effect of the Concentration of Tetra-Sulphonated Nickel Phthalocyanine

**Figure 8:**
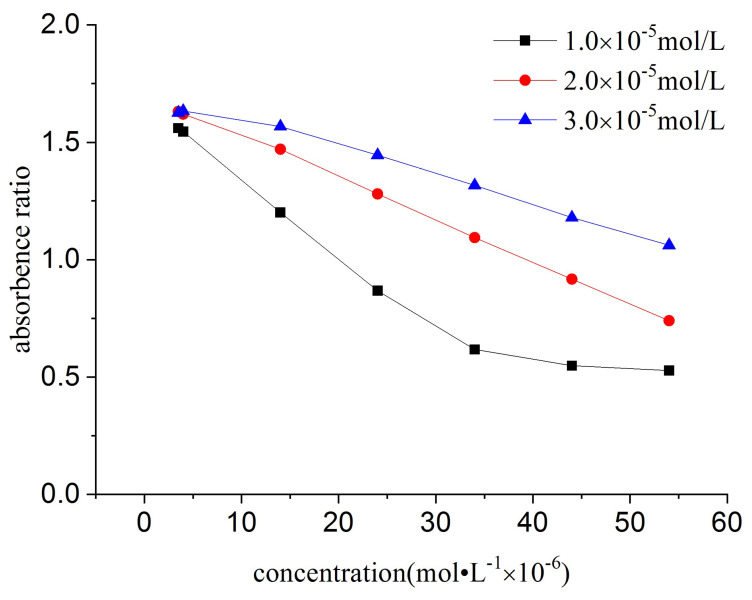
Effect of the concentration of NiS_4_Pc on the calibration curves of CPC.

The calibration curves were constructed using NiS_4_Pc at concentrations of 10.0 μM, 20.0 μM, and 30.0 μM, respectively. It could be seen that the linear range of the calibration graph was shortened at low concentrations of NiS_4_Pc (10.0 μM, Figure 8). The slope of the curve at 10.0 μM was smaller than that at 20.0 μM. Thus, 20.0 μM was finally chosen for the usage of NiS_4_Pc, taking both sensitivity and linear range into account.

### 2.4. Averaging of Calibration Curves

**Figure 9:**
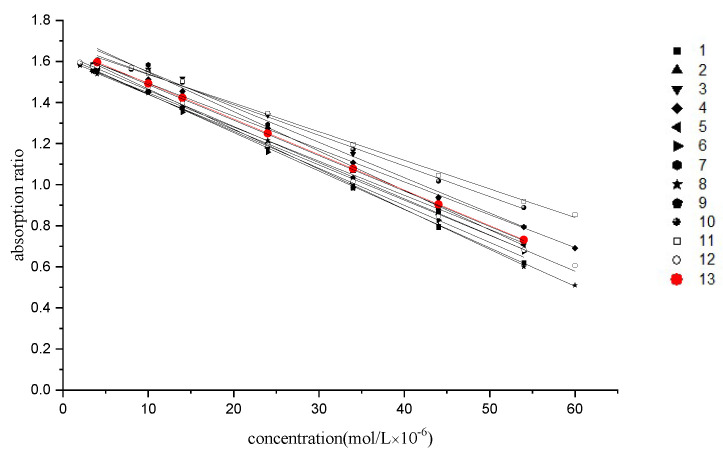
Calibration curves of twelve sorts of cationic surfactants and their mean curves.

Under optimal conditions, the calibration curves of twelve sorts of cationic surfactants were constructed. It can be seen that the calibration curves are close to each other, indicating that NiS_4_Pc responded to these cationic surfactants similarly. This finding implies that the present method can be used for the determination of a single cationic surfactant, as well as for the determination of the total cationic surfactants. These calibration curves were averaged to obtain a mean calibration curve (Figure 9, red line), which is more accurate when the present method is used in a practical application for the determination of the total amounts of cationic surfactants. The linear parameters of the twelve calibration curves and the mean line are summarized in Table 2.

The numbers 1–13 refer to the calibration curves of octadecylpyridinium chloride, hexadecylpyridinium chloride, tetradecylpyridinium chloride, hexadecylpyridinium bromide, hexadecyltrimethylammonium chloride, hexadecyltrimethylammonium bromide, tetradecyltrimethylammonium bromide, hexadecyldimethylbenzylammonium chloride, tetradecyldimethylbenzylammonium chloride, dodecyldimethylbenzylammonium chloride, dodecydimethylbenzylammonium bromide, and hexadecyldimethylethylammonium bromide and the mean calibration curve, respectively.

### 2.5. Interference of Foreign Substances

The interference of foreign substances normally encountered in the detection of cationic surfactants was tested, and the results are given in Table 3. The substances tested showed a slight degree of interference (positive or negative deviation). Usually, a deviation of ±10% is acceptable for an analytical method. It can be seen that most of the substances tested showed acceptable degrees of interference except for dodecyltrimethylammonium bromide.

### 2.6. Determination of Real Samples

The proposed method has been applied to the quantitative analysis of CPC in a commercial product (cetylpyridinium chloride mouthwash, with a CPC content of 0.1%). The concentrations of CPC measured by this method were compared with real values. The analytical results are listed in Table 4.

## 3. Materials and Methods

### 3.1. Equipment and Reagents

The instruments used in this study include UV-Vis spectrophotometers (Evolution220, Thermo Fisher Scientific, Waltham, MA, USA; Lamda 25, Perkin Elmer, Waltham, MA, USA), a 1 cm quartz cuvette, a pH meter (Orion Star A211, Thermo Scientific, USA), an electronic analytical balance (BS124S, Beijing Sartorius Instrument System Co., Ltd., Beijing, China), and an OMNI laboratory ultra-pure water system (Research Scientific Instruments Co., Ltd., Xiamen, China).

Tetra-sulphonated nickel phthalocyanine (NiS_4_Pc), tetra-sulfonated zinc phthalocyanine (ZnS_4_Pc), tetra-sulfonated cobalt phthalocyanine (CoS_4_Pc), and tetra-sulfonated iron phthalocyanine (FeS_4_Pc) were purchased from J&K Scientific Co., Ltd., Beijing, China. The other reagents used include octadecylpyridinium chloride, cetylpyridinium chloride hydrate, tetradecylpyridine chloride, cetylpyridinium bromide (Hefei Xinbiaoxin Chemical Co., Ltd., Hefei, China), cetrimonium chloride, cetrimonium bromide, tetradecyltrimethylammonium bromide (Tianjin Guangfu Fine Chemical Research Institute, Tianjin, China), cetyldimethylbenzylammonium chloride, benzyldimethyltetradecylammonium chloride, benzyldimethyldodecylammonium chloride, benzyldimethyldodecylammonium bromide (Sinopharm Chemical Reagent Co., Ltd., Beijing, China), and cetyldimethylethylammonium bromide (Sangon Biotech Co., Ltd., Shanghai, China).

The concentration of all metal phthalocyanine stock solutions was 1.0 × 10^−2^ mol L^−1^. They were stored at 4 °C and diluted to 1.0 × 10^−3^ mol L^−1^ when used. The concentration of the stock solutions of all cationic surfactants was 1.0 × 10^−2^ mol L^−1^, diluted to 1.0 × 10^−4^ mol L^−1^ when used. Phosphate buffers with a broad-range pH were prepared by mixing stock solutions I (0.10 mol L^−1^ hydrochloric acid), II (1/15 mol L^−1^ potassium dihydrogen phosphate), III (1/15 mol L^−1^ disodium hydrogen phosphate), and IV (1/15 mol L^−1^ sodium phosphate). The buffer with a pH between 1.0 and 4.0 contains stock solutions I and II; the buffer with a pH between 5.0 and 7.0 contains stock solutions II and III; the buffer with a pH between 8.0 and 11.0 contains stock solutions II and IV. The hydrochloric acid–potassium chloride buffer was prepared by mixing 25 mL of 0.20 molL^−1^ potassium chloride solution with hydrochloric acid (0.20 mol L^−1^) and diluting the mixed solution with water to 100.0 mL.

All of the reagents used were of analytical grade, and high-purity water was used throughout.

### 3.2. Experimental Methods

To a 5 mL plastic centrifuge tube, reagents were added in the following sequence: water, a wide-range phosphate buffer (300 μL, pH 1.0), the cationic surfactant solution, and the tertra-sulfonated metal phthalocyanine solution (60 μL, 1.0 × 10^−3^ mol L^−1^). The final volume was 3.0 mL. The solution was mixed and kept at room temperature for 5 min. The absorption spectra were recorded using a spectrophotometer. The absorbance of the solution at 625 nm (dimer) was denoted as A_1_, and the absorbance at 659 nm (monomer) as A_2_. The absorbance ratio was calculated by R = A_1_/A_2_.

## 4. Conclusions

In this study, screening experiments on the absorption spectra of metal phthalocyanines revealed that tetra-sulphonated nickel phthalocyanine (NiS_4_Pc) responded significantly to cationic surfactants tailing long carbon chains containing more than 12 carbon atoms, while showing no response to cationic surfactants with carbon chains of fewer than 12 carbon atoms, indicating a typical structure–activity relationship. Studies of the mechanism of this phenomenon indicate that this specific spectral response behavior could be attributed to the dramatic influence of cationic surfactants whose molecular structures matched with NiS_4_Pc, shifting the self-association equilibrium of NiS_4_Pc. According to the above findings, we established a new method for the quantitative analysis of long carbon chain cationic surfactants using NiS_4_Pc as an optical probe. Because the response behavior of NiS_4_Pc is highly similar to cationic surfactants, this method can be used for the determination of a single cationic surfactant, as well as for the determination of the total cationic surfactants. NiS_4_Pc is a water-soluble optical probe that can be used in a total aqueous phase. Therefore, this method is not only selective but also easy and fast to operate, without the use of any organic solvents. This study provides new ideas for the development of optical probes based on the self-association of phthalocyanine compounds and exhibits a novel application of them in analytical sciences.

## Figures and Tables

**Table 1:** Effect of pH on the linearity of calibration curves of CPC.

pH	Selection of Wavelength Pairs	Linear Equation	Linear Range (μM)	r
1.0	625/659	y = −0.0183x + 1.6921	1.0–54.0	0.9987
2.0	625/659	y = −0.0197x + 1.6150	0.2–44.0	0.9982
3.0	620/659	y = −0.0215x + 1.6232	1.0–44.0	0.9973
4.0	620/659	y = −0.0195x + 1.5785	4.0–54.0	0.9908
5.0	620/659	y = −0.0199x + 1.5822	4.0–54.0	0.9884
6.0	621/660	y = −0.0198x + 1.5974	4.0–54.0	0.9886
7.0	622/659	y = −0.0176x + 1.5619	4.0–54.0	0.9850
8.0	620/659	y = −0.0180x + 1.5832	4.0–54.0	0.9925
9.0	620/659	y = −0.0182x + 1.5860	4.0–54.0	0.9898
10.0	620/658	y = −0.0178x + 1.5729	4.0–54.0	0.9894
11.0	620/659	y = −0.0177x + 1.5857	4.0–54.0	0.9923

**Table 2:** Regression equations of twelve sorts of cationic surfactants and the mean calibration curve.

No.	Cationic Surfactant	Regression Equation	Linear Range (μM)	Correlation
1	octadecylpyridinium chloride	y = 1.66 − 0.019x	3.5–54.0	0.9991
2	cetylpyridinium chloride	y = 1.65 − 0.017x	3.5–54.0	0.9991
3	tetradecylpyridinium chloride	y = 1.75 − 0.018x	10–54.0	0.9976
4	hexadecylpyridinium bromide	y = 1.65 − 0.016x	3.5–60.0	0.9990
5	hexadecyltrimethylammonium chloride	y = 1.61 − 0.017x	3.5–54.0	0.9988
6	hexadecyltrimethylammonium bromide	y = 1.62 − 0.018x	3.5–54.0	0.9982
7	tetradecyltrimethylammonium bromide	y = 1.77 − 0.020x	10–54.0	0.9984
8	hexadecyldimethylbenzylammonium chloride	y = 1.62 − 0.019x	2.0–60.0	0.9990
9	tetradecyldimethylbenzylammonium chloride	y = 1.61 − 0.017x	2.0–54.0	0.9992
10	dodecyldimethylbenzylammonium chloride	y = 1.69 − 0.015x	4.0–54.0	0.9982
11	dodecydimethylbenzylammonium bromide	y = 1.67 − 0.014x	4.0–60.0	0.9984
12	dexadecyldimethylethylammonium bromide	y = 1.64 − 0.018x	2.0–60.0	0.9990
13	mean calibration curve	y = 1.67 − 0.017x	4.0–54.0	0.9987
	Limit of Detection (LOD)	3 × 10^−6^ μM		

**Table 3:** Interference of foreign substances on the detection of CPC ([CPC] = 30.0 μM).

Substance	Concentration (mol/L)	Relative Error (%)	Substance	Concentration (mol/L)	Relative Error (%)
NaIO_4_	1.5 × 10^−4^	−5.98	KBr	3 × 10^−2^	−6.29
Na_2_S_2_O_3_	3 × 10^−4^	0.36	NH_4_Cl	3 × 10^−2^	−2.04
CoCl_2_	3 × 10^−4^	4.92	NaH_2_PO_4_	3 × 10^−2^	−3.79
Pb (NO_3_) _2_	3 × 10^−3^	7.58	Urea	3 × 10^−2^	1.79
BaCl_2_	3 × 10^−3^	−4.37	Boric acid	3 × 10^−2^	0.86
CaCl_2_	3 × 10^−3^	4.58	Pyridine	3 × 10^−3^	−3.57
CuSO_4_	3 × 10^−3^	8.62	Decyltrimethylammonium Bromide	3 × 10^−5^	−2.70
NaF	3 × 10^−3^	−1.19	Benzyltributylammonium chloride	3 × 10^−4^	−0.18
Na_3_PO_4_	3 × 10^−3^	−0.07	Tetrabutylammonium bromide	3 × 10^−3^	−0.52
NaI	3 × 10^−3^	1.94	Tetramethylammonium bromide	3 × 10^−2^	−6.10
EDTA	3 × 10^−3^	−3.71	Tetraethylammonium bromide	3 × 10^−2^	−1.00
Na_2_CO_3_	3 × 10^−3^	−1.67	Tetramethylammonium bromide	3 × 10^−2^	−1.08
NaHCO_3_	3 × 10^−3^	−3.39	Dodecyltrimethylammonium bromide	3 × 10^−5^	−12.69
NaClO_4_	3 × 10^−3^	−0.81			
AgNO_3_	3 × 10^−3^	−2.89			
0 (without interference	0	0.17			

**Table 4:** Analytical results of real samples.

Concentrations of CPC in Real Samples (μM) *	Absorbance Ratio (A_625 nm_/A_659 nm_)	Concentrations Measured by This Method (μM)	Relative Error (%)	RSD (%)
4.65	1.58	4.68	0.64	2.84
18.60	1.35	18.42	−0.97	1.07
27.90	1.15	29.86	7.02	0.26
37.20	1.02	37.53	0.90	0.46
46.50	0.81	49.31	6.03	0.42

*: Calculated by the labeled quantity.

## Data Availability

Data are contained within the article and Supplementary Materials.

## Supplementary Materials

The following supporting information can be downloaded at: https://www.mdpi.com/article/10.3390/molecules30214184/s1, Figure S1: Influence of ion strength on the calibration curve; Figure S2: Absorption spectra of NiS_4_Pc in the presence of cationic surfactants with carbon chains containing carbons less twelve; Figure S3: Absorption spectra of NiS_4_Pc in the presence of cationic surfactants tailing a long carbon chains.
