# Supplementary material for: A Phthalocyanine Optical Probe Responding to Cationic Surfactants Containing Long Carbon Chains with High Selectivity in Total Water Phase and Its Applications
Source: Molecules. 2025 Oct 26;30(21):4184. doi: 10.3390/molecules30214184 (PMC12610040; doi:10.3390/molecules30214184)
Supplement: Supplementary file 1 [file molecules-30-04184-s001.zip › molecules-3897165-supplementary.pdf]

Supplementary Materials

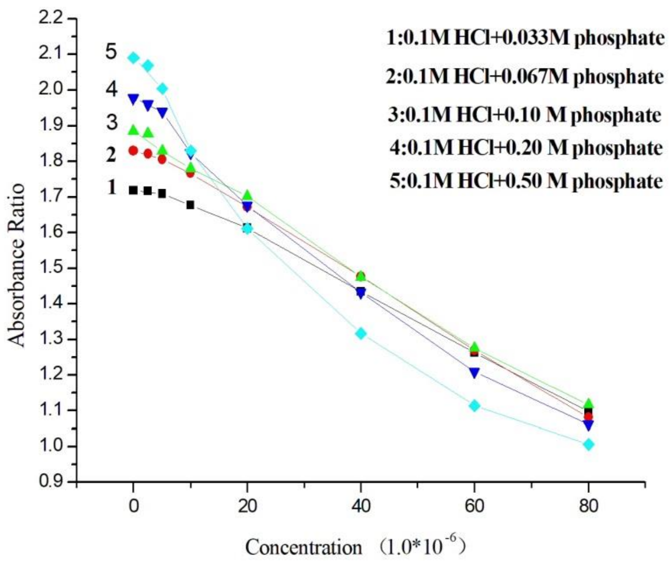

Figure S1 Influence of ion strength on the calibration curve

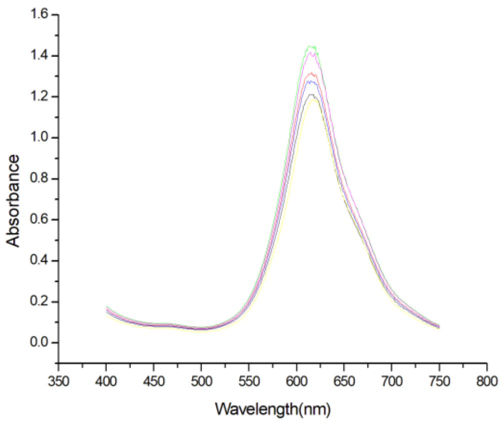

Figure S2. Absorption spectra of NiS_4Pc in the presence of cationic surfactants with carbon chains containing carbons less twelve. The cationic surfactants tested were tetra Methyl Ammonium Bromide, tetraethylammonium bromide, tetrapropylammonium bromide, tetrabutylammonium bromide, tetraheptylammonium bromide, tetra-n-octylammonium bromide, and tecyltrimethylammonium bromide.

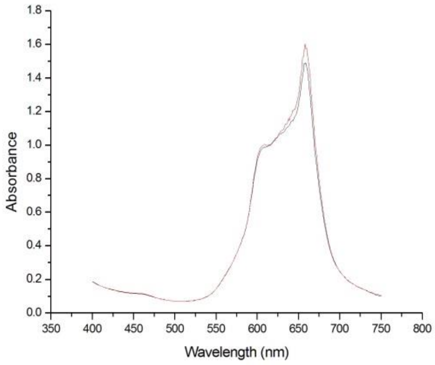

Figure S3. Absorption spectra of NiS_4Pc in the presence of cationic surfactants tailing a long carbon chains. The cationic surfactants tested were tetradecyltrimethylammonium bromide and hexadecyltrimethylammonium bromide.
